# Ablation of the circadian rhythm protein CACNA2D3 impairs primordial follicle assembly in the mouse ovary

**DOI:** 10.1002/ctm2.1467

**Published:** 2023-11-06

**Authors:** Wen‐Xiang Liu, Xin‐Xiang Xie, Hong‐Chen Yan, Francesca Gioia Klinger, Maria Dri, Massimo De Felici, Wei Shen, Bin‐Bin Wang, Shun‐Feng Cheng

**Affiliations:** ^1^ College of Life Sciences, Institute of Reproductive Sciences Qingdao Agricultural University Qingdao China; ^2^ Saint Camillus International University of Health Sciences Rome Italy; ^3^ Department of Biomedicine and Prevention University of Rome Tor Vergata Rome Italy; ^4^ Center for Genetics National Research Institute for Family Planning Beijing China

To the Editor:

The female mammalian reproductive system relies on a complex coordination of central and peripheral oscillators, with the suprachiasmatic nucleus (SCN) serving as the primary pacemaker.^1^ Disruptions in circadian regulation can have detrimental effects on overall health and reproductive processes.^2^ The absence of *Cacna2d3*, a subtype 3 of α2δ proteins expressed in the SCN, degrades clock neurons, affecting the sustainability of central oscillators.^3^ However, the role of *Cacna2d3* in reproduction remains unknown. In this study, we employed single‐cell RNA sequencing (scRNA‐seq) to investigate the impact of *Cacna2d3* knockout (KO) on ovarian development and fertility in female mice.

When mated with wild‐type (WT) males, *Cacna2d3* KO homozygous females were fertile, but at the mean old age (20 weeks), they delivered a significant lower number of pups in comparison to WT females (Figure 1E,F). Moreover, we observed that KO females had much smaller ovaries than WT counterparts already at 3‐week‐old (Figure 1G), and that such KO ovaries possessed a significant lower number of follicles than WT, in particular of primordial follicle (PF) (Figure 1H–J).

**FIGURE 1:**
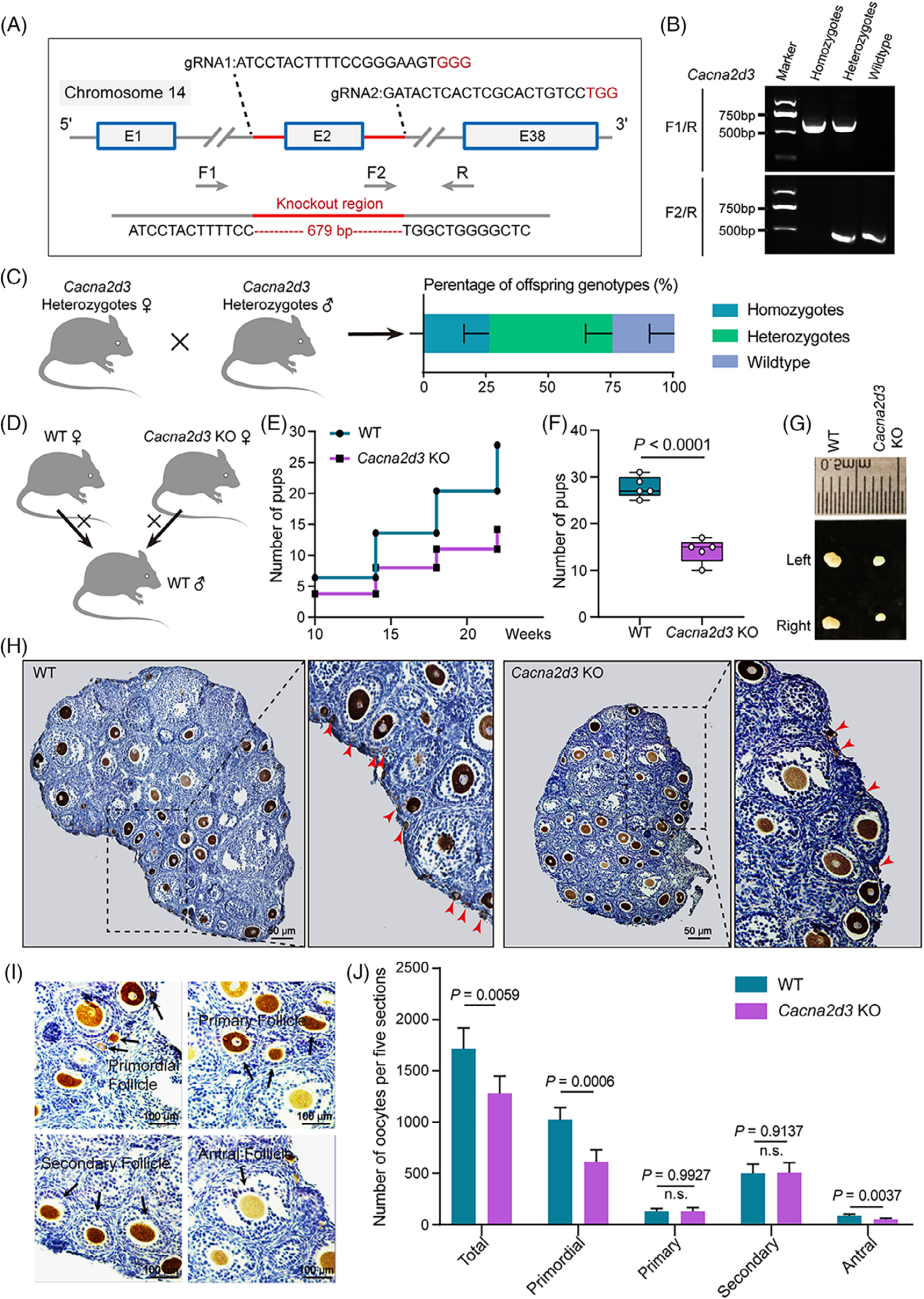
Generation and characterisation of *Cacna2d3* KO mouse females. (A) Gene targeting strategy for CRISPR/Cas9‐based *Cacna2d3* KO. F, forward; R, reverse; gRNA, guide RNA. (B) RT‐PCR screening results using tail genomic DNA as the template. (C) The offspring genotypes were counted after mating between heterozygotes. (D) Schematic diagram of WT males mating with WT fand *Cacna2d3* KO females, respectively. (E and F) Cumulative numbers of pups per WT and *Cacna2d3* KO females. Biologically independent repeats. (G) Representative images of 21 dpp WT and *Cacna2d3* KO ovaries. (H) Representative immunohistochemistry (IHC) of DDX4‐positive oocytes (dark brown) in tissue sections of ovaries from 21 dpp WT and *Cacna2d3* KO females. The red arrow indicates the primordial follicle. Scale bar: 50 μm. (I) Higher magnification of (H) showing different classes of follicles. Scale bar: 100 μm. (J) Quantification of the total, primordial, primary, secondary, and antral follicle numbers in 21 dpp WT and *Cacna2d3* KO ovaries. Biologically independent repeats.

In mice, the majority of the ovarian PF stockpile is established shortly after birth through the germline cyst breakdown (Figure 2A).^4^ To determine the cause of reduced PF numbers in the ovaries of 3‐week‐old *Cacna2d3* KO female mice, we examined 3 days post‐partum (dpp) ovaries for potential defects in PF assembly. Analysis of tissue sections stained for the oocyte‐specific cytoplasmic protein DDX4 revealed a significantly lower percentage of oocytes individually enclosed into follicles in the KO ovaries compared to WT ovaries (Figure 2B–D). We performed scRNA‐seq analyses on dissociated 3dpp ovarian from WT and *Cacna2d3* KO females, resulting in 16 705 ovarian cells (Figure 2E). By applying data filtering and Uniform Manifold Approximation and Projection (UMAP) analysis, we identified 14 cell clusters and classified the cells into six types based on previous studies and the expression of specific genes,^4^ including oocytes, pregranulosa (PG) cells, stromal cells, erythrocytes, immune cells, and endothelial cells (Figure 2F; Figures S1 and S2; Table S1). Considering that PF formation requires essential interaction between oocytes and PG cells, these clusters were extracted for further analysis (Figure S3A). We found that in KO ovaries, both cell types showed substantial reduced levels of mRNAs for circadian rhythmic related genes (Figure S3B,C), while *Cacna2d3* mRNAs were not detectable either in WT or KO ovaries (not shown).

**FIGURE 2:**
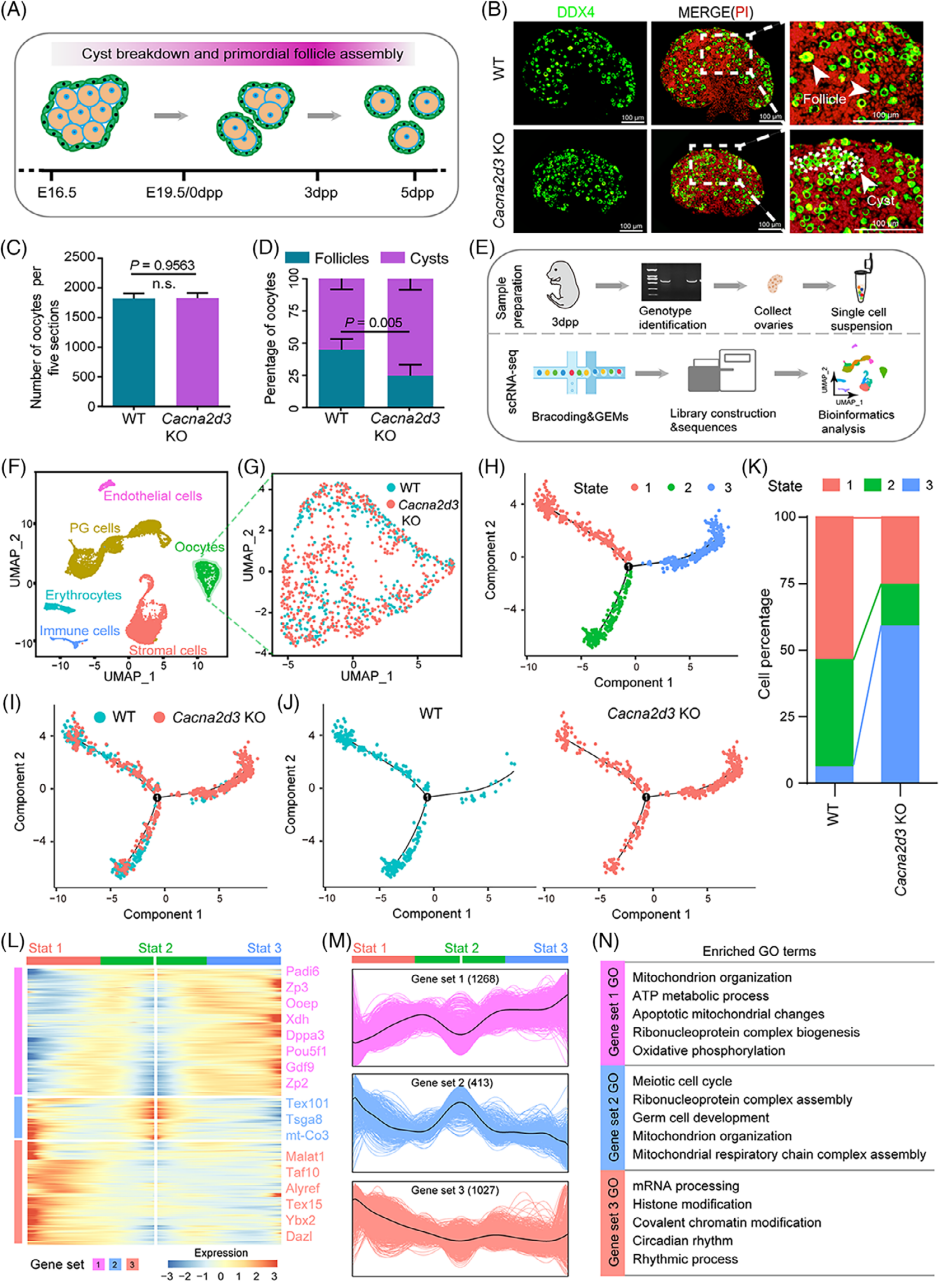
Characterising primordial follicle assembly and transcriptome dynamics of *Cacna2d3* KO oocytes from 3 dpp ovaries. (A) Schematic diagram of germ cell cyst breakdown and primordial follicle assembly in function of embryonal (E) and post‐partum (dpp) age. (B) Representative images of germ cell cyst breakdown and primordial follicle assembly in tissue sections of WT and *Cacna2d3* KO 3 dpp ovaries; DDX4 positivity identify oocytes (green) in cysts or singly enclosed in PFs while nuclei of all cells were counterstained by propidium iodide (PI, red). Scale bar: 100 μm. (C) Total number of oocytes. Biologically independent repeats. (D) Percentages of oocytes in cysts or within PFs. (E) Schematic diagram of sample preparation for single‐cell RNA sequence (scRNA‐seq). Biologically independent repeats. (F) The six main ovarian cell types identified using UMAP plot. (G) Extraction of oocyte clusters from the UMAP map. (H) Single‐cell pseudotime developmental trajectory of the oocyte populations showing three states and one branching point. (I and J) Pseudotime trajectory of oocytes from both WT and *Cacna2d3* KO ovaries. (K) Percentage distribution of the oocytes into the three states. (L) Pseudotime ordered heatmap of three DEGs sets between two obvious fates from State 2 to State 1 or State 3. (M) Graph showing the expression of the three DEGs sets in the pseudotime trajectory. (N) The enrichment of GO terms in gene sets 1, 2 and 3.

The developmental pseudotime trajectory of the oocyte populations indicated an abnormal developmental deviation for the most part of oocytes in KO ovaries (Figure 2G–K). Further analyses allowed allocating three gene sets with distinct expression patterns along the pseudotime trajectory (Figure 2L,M; Table S2). Gene ontology (GO) analysis revealed significant changes in genes associated with energy metabolism and circadian rhythm following *Cacna2d3* KO (Figure 2N).

Subsequently, PG cells were extracted as clusters (Figure S4A) and categorised as either epithelial generated PG (EPG) cells or bipotential generated PG (BPG) cells, based on previous studies^5^ (Figure S4A–J; Figure S5A,B). BPG or EPG cells were divided into three cell states, and the expression of which changed significantly along with cell trajectory, respectively (Figure S5C–J). Interestingly, in the representative gene sets of altered cellular states, GO terms related to energy metabolism were also significantly enriched in both BPG and EPG cells (Figure S5K,L; Tables S3 and S4).

Further transcriptome analyses inferred several additional differences between WT and *Cacna2d3* KO oocytes and PG cells. Notably, *Cacna2d3* knockout resulted in elevated expression of genes related to oxidative phosphorylation (OXPHOS) signalling pathways in oocytes, enrichment of OXPHOS and reactive oxygen species (ROS) pathways, increased expression of DNA repair‐related genes, and decreased expression of germ cell‐specific genes (Figure 3A; Figure S6A–E). Furthermore, higher expression of γ‐H2AX^6^ on tissue sections of ovaries indicated increased DNA damage in KO oocytes (Figure 3B–D). The analysis of WT and *Cacna2d3* KO BPG and EPG cells showed that the majority of changes in GO terms were shared between these subpopulations, likely due to the presence of common differentially expressed genes (DEGs) (Figure S7A–D; Table S6). Moreover, similar to oocytes, both BPG and EPG cells exhibited enrichment in OXPHOS and ROS processes (Figure S7E,F). Moreover, *Cacna2d3* KO ovaries exhibited decreased expression of germ cell‐specific genes and increased expression of key genes associated with OXPHOS signalling (Figure 3E–G; Figure S8). Correspondingly, ROS level, *Sod1* expression level, mitochondrial permeability transition pore complex expression level and GSH/GSSG ratio assays^7^ further demonstrated DNA damage in oocytes as likely consequence of ROS increase (Figure 3H–K; Figure S9A,B). There was a reduction in the expression of genes related to PI3K‐AKT and MAPK signalling pathways in the KO ovaries (Figure S9C,D).

**FIGURE 3:**
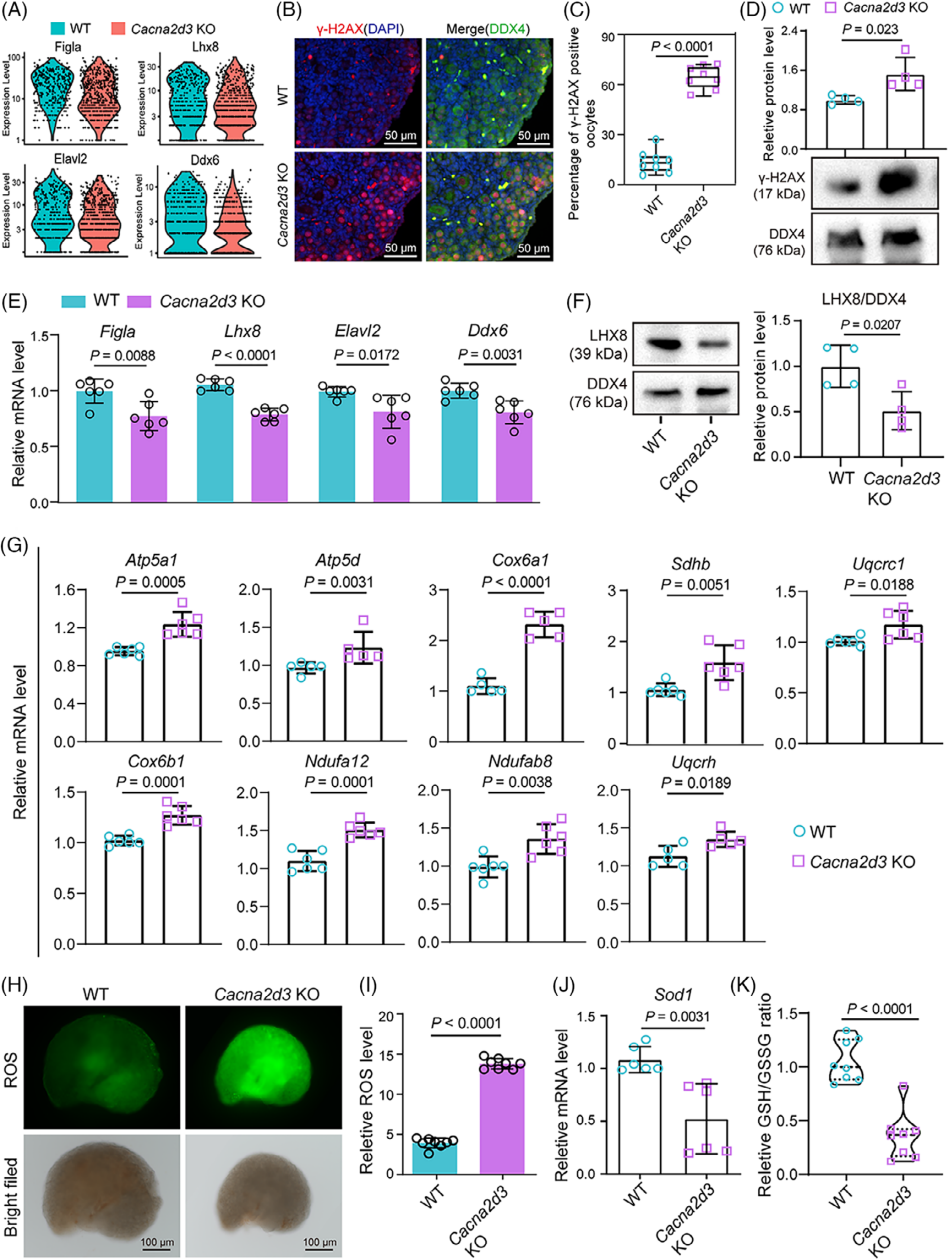
Impact of *Cacna2d3* deletion on oocyte‐specific gene expression, DNA damage response and oxidative stress in mouse ovaries. (A) Violin plots of the expression level of oocyte‐specific expression gene in WT and *Cacna2d3* KO mice. (B) Representative IF images of γ‐H2AX (red) and DDX4 (green) double staining on tissue sections of WT and *Cacna2d3* KO ovaries. Scale bar: 50 μm. (C) Percentage of γ‐H2AX‐positive oocytes in WT and *Cacna2d3* KO ovaries. Biologically independent repeats. (D) WB and quantification of γ‐H2AX protein in WT and *Cacna2d3* KO ovaries. Biologically independent repeats. (E) RT‐qPCR for *Figla*, *Lhx8*, *Elavl2* and *Ddx6* mRNA performed on tissue samples of the WT and *Cacna2d3* KO 3 dpp ovaries. Biologically independent repeats. (F) Representative WB for LHX8 on WT and *Cacna2d3* KO ovaries. Biologically independent repeats. (G) RT‐qPCR for mRNA of OXPHOS signalling pathway genes performed on tissue samples of WT and *Cacna2d3* KO ovaries. Biologically independent repeats. (H) Representative pictures showing ROS production in WT and *Cacna2d3* KO ovaries. Scale bar: 100 μm. (I) Quantification of ROS amount in WT and *Cacna2d3* KO ovaries. Biologically independent repeats. (J) RT‐qPCR for *Sod1* mRNA performed on tissue samples of WT and *Cacna2d3* KO ovaries. Biologically independent repeats. (K) Relative GSH/GSSG ratio in WT and *Cacna2d3* KO ovaries. Biologically independent repeats.

Next, we focused on cell–cell communication, in particular between oocytes and PG cells.^4^, ^8^ CellChat analysis revealed that the interaction strength was significantly decreased due to *Cacna2d3* ablation (Figure 4A; Figure S10A,B). *Cacna2d3* KO led to altered signalling patterns among ovarian cells, with significantly reduced interaction strength in oocytes and PG cells (Figure 4B,C; Figure S10C,D). Subsequently, we identified altered ligand–receptor pairs between oocytes and PG cells, including changes in eight PG cell ligand and oocyte receptor pairs, as well as two oocyte ligand and PG cell receptor pairs, resulting from *Cacna2d3* ablation (Figure 4D,E). Furthermore, violin plots and RT‐qPCR showed that *Cacna2d3* KO significantly downregulated the expression of all tested ligands or receptors, especially *Kitl*‐*Kit*, which were predominantly expressed in PG cells and oocytes, respectively (Figure 4F–H; Figure S10E). Meanwhile, *Kit*‐siRNA treatment in newborn ovaries led to reduced c‐KIT protein levels, and a decreased number of oocytes, with a lower percentage enclosed in PFs (Figure 4I–L).

**FIGURE 4:**
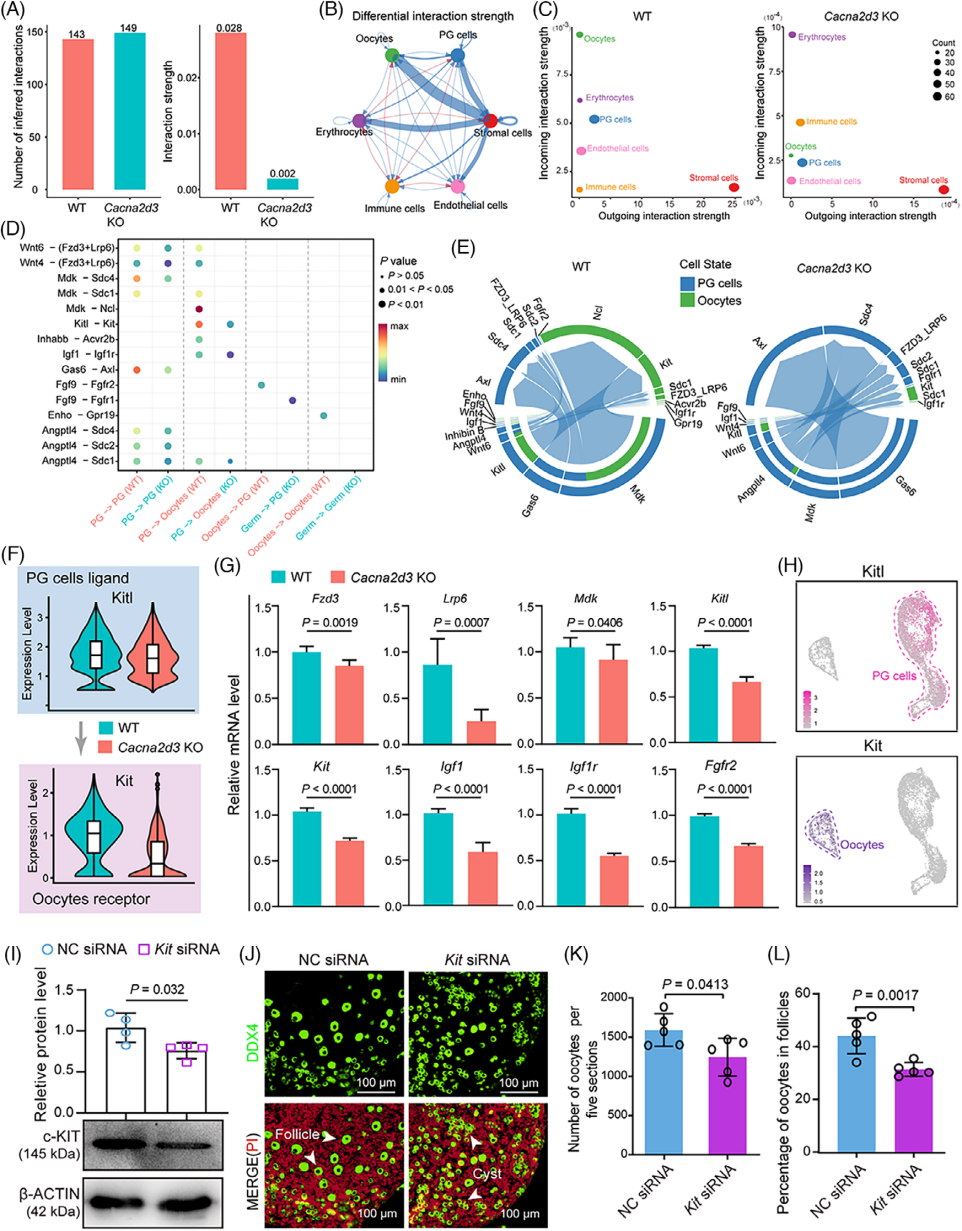
*Cacna2d3* KO impairs communication between oocytes and PG cells in 3 dpp ovaries. (A) Bar chart showing the number of inferred global interactions (left) and their strength (right) in WT and *Cacna2d3* KO 3 dpp ovaries predicted by CellChat analysis. (B) Differential interaction strength among the six main ovarian cell populations in WT and *Cacna2d3* KO ovaries; red and blue indicate increase or decrease of strength, respectively, in KO compared with WT ovaries. (C) 2D spatial maps of the intensity of incoming and outgoing interaction strength of six cell population in WT and *Cacna2d3* KO ovaries. (D and E) Dot plot and chord charts of the expression level of differentially upregulated and downregulated signal ligand–receptor pairs in oocyte and PG cell clusters of WT and *Cacna2d3* KO ovaries. (F) Violin plots of the expression level of the oocyte *Kit* receptor and PG cell *Kitl* ligand in WT and *Cacna2d3* KO ovaries. (G) RT‐qPCR for mRNA of ligand and receptor genes in WT and *Cacna2d3* KO ovaries. Biologically independent repeats. (H) Feature plots of *Kit* and *Kitl* expression in oocytes and PG cells. (I) WB and relative quantification of KIT amount in 3 dpp ovary cultured for 3 days in the presence of CTRL (NC siRNA) and *Kit* siRNA. Biologically independent repeats. (J) Representative images of germ cell cyst and PF in sections of ovaries cultured under the indicated conditions. DDX4‐positive oocytes (green); PI‐stained cell nuclei (red). Scale bar: 100 μm. (K) Total number of oocytes. Biologically independent repeats. (L) Percentage of oocytes singly enclosed into PFs in ovaries after 3 days under the indicated conditions. Biologically independent repeats.

In conclusion, we found that *Cacna2d3* KO mice had significantly reduced ovarian follicles, and this might result from impaired PF formation in neonatal ovaries consequent to alteration of energy homeostasis and cell–cell communications in oocytes and PG cells (Figure S11). Further investigation is needed to elucidate the underlying mechanisms and identify potential therapeutic targets for the treatment of primary ovarian insufficiency associated with early‐age circadian rhythm disruption.

## CONFLICT OF INTEREST STATEMENT

The authors declare they have no conflicts of interest.

## FUNDING INFORMATION

National Natural Science Foundation of China, Grant Numbers: 32072734 and 32100683; Shandong Taishan Scholars Construction Foundation, Grant Number: ts20190946; Qingdao Science and Technology Benefit the People Demonstration and Guidance Special Project, Grant Number: 23‐1‐3‐3‐zyyd‐nsh; CAMS Innovation Fund for Medical Sciences, Grant Number: 2018‐I2M‐1‐004.

## Supporting information

Supporting Information

Supporting Information

Supporting Information

Supporting Information

Supporting Information

Supporting Information

Supporting Information

Supporting Information

Supporting Information

## Data Availability

The raw data of scRNA‐seq for ovarian tissues following *Cacna2d3* KO have been deposited in the Genome Sequence Archive with the accession number: CRA005498.
