# Supplementary material for: Ablation of the circadian rhythm protein CACNA2D3 impairs primordial follicle assembly in the mouse ovary
Source: Clin Transl Med. 2023 Nov 6;13(11):e1467. doi: 10.1002/ctm2.1467 (PMC10626498; doi:10.1002/ctm2.1467)
Supplement: Supplementary file 7 — Supporting Information [file CTM2-13-e1467-s002.docx]

**Table S7. Antibodies used in this paper.**

| **Primary antibodies** | **Vendor** | **Dilution** | Source |
| --- | --- | --- | --- |
| DDX4 (IF/WB/IHC)  DDX4 (IF)  LHX8 (WB)  β-Actin (WB)  γ-H2AX(IF/WB) | Abcam (ab13840)  Abcam (ab27591)  Abcam (ab137036)  Sigma (A1974)  Abcam (ab26350) | 1:200/1:1000/1:200  1:200  1:800  1:1000  1:200/1:1000 | Rabbit  Mouse  Rabbit  Mouse  Rabbit |
| **Secondary antibodies**  CY3-conjugated goat anti-rabbit(IF)  FITC-conjugated goat anti-rabbit(IF)  HRP-conjugated goat anti- Mouse IgG(WB)  HRP-conjugated goat anti- rabbit IgG(WB) | Beyotime (A0516)  Beyotime (A0562)  Beyotime (A0216)  Beyotime (A0258) | 1:200  1:200  1:1000  1:1000 | Goat  Goat  Goat  Goat |
