# Supplementary material for: Ablation of the circadian rhythm protein CACNA2D3 impairs primordial follicle assembly in the mouse ovary
Source: Clin Transl Med. 2023 Nov 6;13(11):e1467. doi: 10.1002/ctm2.1467 (PMC10626498; doi:10.1002/ctm2.1467)
Supplement: Supplementary file 8 — Supporting Information [file CTM2-13-e1467-s001.docx]

**Table S8. Primers Used for Quantitative RT-PCR.**

| **Genes** | **Genbank** | **Forward primer sequence** | **Reverse primer sequence** | **Product Length (bp)** |
| --- | --- | --- | --- | --- |
| *Lhx8*  *Ddx6*  *Figla*  *Elavl2*  *Atp5a1*  *Atp5d*  *Cox6a1*  *Cox6b1*  *Ndufa12*  *Ndufa8*  *Sdhb*  *Uqcrc1*  *Uqcrh*  *Sod1*  *Fzd3*  *Lrp6*  *Mdk*  *Kitl*  *Kit*  *Igf1*  *Igf1r*  *Fgfr2*  *Clock*  *Per3*  *Ddx4*  *Gapdh* | NC_000069.5  [NM_181324](http://www.ncbi.nlm.nih.gov/entrez/query.fcgi?cmd=Search&db=Nucleotide&term=NM_181324)  [NM_012013](http://www.ncbi.nlm.nih.gov/entrez/query.fcgi?cmd=Search&db=Nucleotide&term=NM_012013)  [NM_207685](http://www.ncbi.nlm.nih.gov/entrez/query.fcgi?cmd=Search&db=Nucleotide&term=NM_207685)  [NM_007505](http://www.ncbi.nlm.nih.gov/entrez/query.fcgi?cmd=Search&db=Nucleotide&term=NM_007505)  [NM_025313](http://www.ncbi.nlm.nih.gov/entrez/query.fcgi?cmd=Search&db=Nucleotide&term=NM_025313)  [NM_007748](http://www.ncbi.nlm.nih.gov/entrez/query.fcgi?cmd=Search&db=Nucleotide&term=NM_007748)  [NM_025628](http://www.ncbi.nlm.nih.gov/entrez/query.fcgi?cmd=Search&db=Nucleotide&term=NM_025628)  [NM_025551](http://www.ncbi.nlm.nih.gov/entrez/query.fcgi?cmd=Search&db=Nucleotide&term=NM_025551)  [NM_026703](http://www.ncbi.nlm.nih.gov/entrez/query.fcgi?cmd=Search&db=Nucleotide&term=NM_026703)  [NM_023374](http://www.ncbi.nlm.nih.gov/entrez/query.fcgi?cmd=Search&db=Nucleotide&term=NM_023374)  [NM_025407](http://www.ncbi.nlm.nih.gov/entrez/query.fcgi?cmd=Search&db=Nucleotide&term=NM_025407)  [NM_025641](http://www.ncbi.nlm.nih.gov/entrez/query.fcgi?cmd=Search&db=Nucleotide&term=NM_025641)  [NM_011434](http://www.ncbi.nlm.nih.gov/entrez/query.fcgi?cmd=Search&db=Nucleotide&term=NM_011434)  [NM_021458](http://www.ncbi.nlm.nih.gov/entrez/query.fcgi?cmd=Search&db=Nucleotide&term=NM_021458)  [NM_008514](http://www.ncbi.nlm.nih.gov/entrez/query.fcgi?cmd=Search&db=Nucleotide&term=NM_008514)  [NM_001012335](http://www.ncbi.nlm.nih.gov/entrez/query.fcgi?cmd=Search&db=Nucleotide&term=NM_001012335)  [NM_013598](http://www.ncbi.nlm.nih.gov/entrez/query.fcgi?cmd=Search&db=Nucleotide&term=NM_013598)  [NM_001122733](http://www.ncbi.nlm.nih.gov/entrez/query.fcgi?cmd=Search&db=Nucleotide&term=NM_001122733)  [NM_001111274](http://www.ncbi.nlm.nih.gov/entrez/query.fcgi?cmd=Search&db=Nucleotide&term=NM_001111274)  [NM_010513](http://www.ncbi.nlm.nih.gov/entrez/query.fcgi?cmd=Search&db=Nucleotide&term=NM_010513)  [NM_201601](http://www.ncbi.nlm.nih.gov/entrez/query.fcgi?cmd=Search&db=Nucleotide&term=NM_201601)  [NM_007715](http://www.ncbi.nlm.nih.gov/entrez/query.fcgi?cmd=Search&db=Nucleotide&term=NM_007715)  [NM_011067](http://www.ncbi.nlm.nih.gov/entrez/query.fcgi?cmd=Search&db=Nucleotide&term=NM_011067)  [NM_010029](http://www.ncbi.nlm.nih.gov/entrez/query.fcgi?cmd=Search&db=Nucleotide&term=NM_010029)  [NM_008084](http://www.ncbi.nlm.nih.gov/entrez/query.fcgi?cmd=Search&db=Nucleotide&term=NM_008084) | CAGTTCGCTCAGGACAACAA  GAACCCTGTTATCATGGGTCTG  CCGCCATCTGTAGGCTCAAG  ACACAGCCAATGGTCCAACC  TCTCCATGCCTCTAACACTCG  TGCTTCAGGCGCGTACATAC  TCAACGTGTTCCTCAAGTCGC  ACTACCTGGACTTCCACCG  ACCGATGGGTCATCTACACCA  GGAGCTGCCAACTCTGGAAG  AATTTGCCATTTACCGATGGGA  AGACCCAGGTCAGCATCTTG  GTGGACCCCCTAACAACAGTG  AACCAGTTGTGTTGTCAGGAC  ATGGCTGTGAGCTGGATTGTC  TTGTTGCTTTATGCAAACAGACG  GAAGAAGGCGCGGTACAATG  GAATCTCCGAAGAGGCCAGAA  GCCACGTCTCAGCCATCTG  GTGAGCCAAAGACACACCCA  GTGGGGGCTCGTGTTTCTC  GCCTCTCGAACAGTATTCTCCT  AGAACTTGGCATTGAAGAGTCTC  AAAAGCACCACGGATACTGGC  GCTTCATCAGATATTGGCGAGT  AGGTCGGTGTGAACGGATTTG | CCTGCAGTTCTGAAACCACA  GCCATTATTGATTGTGCTGGTGT  ACACAGCCGAGTATCTGTATGTA  TTCCCGGAGTCAACTGGTGA  CCAGGTCAACAGACGTGTCAG  CACTTGCTTGACGTTGGCA  AGGGTATGGTTACCGTCTCCC  ACCCATGACACGGGACAGA  TCGTCAGTCATGCAGTGAAGC  CCAGCGGCACAGCATAAAC  AGCATCCAACACCATAGGTCC  GCCGATTCTTTGTTCCCTTGA  CGGGAAGACACGCGATTATCA  CCACCATGTTTCTTAGAGTGAGG  GGCACATCCTCAAGGTTATAGGT  GTTCGTTTAATGGCTTCTTCGC  GAGGTGCAGGGCTTAGTCA  GCTGCAACAGGGGGTAACAT  GTCGCCAGCTTCAACTATTAACT  ACCTCTGATTTTCCGAGTTGC  GATCACCGTGCAGTTTTCCA  ACAGGGTTCATAAGGCATGGG  GTCAGACCCAGAATCTTGGCT  GGGAGGCTGTAGCTTGTCA  GCTTGGAAAACCCTCTGCTT  TGTAGACCATGTAGTTGAGGTCA | 105  142  238  61  101  128  115  112  112  130  104  174  101  139  109  170  63  197  90  68  127  103  67  119  193  123 |
