# Supplementary material for: Ablation of the circadian rhythm protein CACNA2D3 impairs primordial follicle assembly in the mouse ovary
Source: Clin Transl Med. 2023 Nov 6;13(11):e1467. doi: 10.1002/ctm2.1467 (PMC10626498; doi:10.1002/ctm2.1467)
Supplement: Supplementary file 9 — Supporting Information [file CTM2-13-e1467-s008.docx]

**Supplementary Material**

**Supplementary materials and methods**

**1. Animals**

Heterozygous KO *Cacna2d3* mice were generated using CRISPR/Cas9 technology in collaboration with from Cyagen Biosciences Inc (S-KO-01286, Guangzhou, China). Wild-type (WT) C57BL/6 mice were obtained from Vital River Laboratory Animal Technology Co. Ltd (Beijing, China). Mice were housed in a controlled environment with food and water *ad libitum*. All the experiments were approved by national rules and the Qingdao Agricultural University Ethics Committee, China. The generation of homozygous targeted mice was obtained by inter-cross heterozygous targeting mice. (Figure 1A-B). Mating between heterozygotes gave mouse genotypes conformed to Mendelian genetics, which means that *Cacna2d3* KO was not lethal (Figure 1C).

The MiniBEST Universal Genomic DNA Extraction Kit (TaKaRa, 9765, Beijing, China) was used to extract the genome from newborn mouse tail tissue. Primer for forward 1 (F1): 5’-TAAGTGCCTGGTGTTGGGACTC-3’, F2: 5’-CCAAACAAACGTGAGCCCAGAG-3’ and reverse ®: 5’-AACCACATCCATTCGCCTTATCTG-3’, were used to select homozygotes, heterozygotes and WT mice. PCR was carried out using 2 × EasyTag PCR SuperMix (Transgene, AT311-03, Beijing, China) following the manufacturer’s recommendations. The PCR conditions were as follows: 3 minutes at 94 °C; 33 cycles of 0.5 minutes at 94 °C, 0.5 minutes at 60 °C, 0.5 minutes at 65 °C; 10 minutes at 65 °C; and finally a cooling step at 4 °C.

**2. Immunofluorescence (IF)**

IF was performed on paraffin ovary sections ^[1]^. In brief, ovaries of 3 days post-partum (dpp) and 21 dpp were fixed in 4% PFA (Solarbio, P1110, Beijing, China) for 12 hours, followed by dehydration and embedding procedures. The paraffin blocks were cut into serial 5 µm slices and mounted on slides. Following gradient rehydration and antigen retrieval (10 mM sodium citrate buffer at 100 °C for 10 min), sections were treated as follows: blocking (3% BSA and 10% normal goat serum in TBS) at 25 °C for 1 hour; primary antibody (Table S7) incubation at 4 °C overnight; washing with PBST; secondary antibody incubation at 25 °C for 1 hour. Nuclear staining was performed with PI (red) or DAPI (blue). Finally, the images were analyzed with a BX51 microscope.

Primordial follicles (PF) were defined as one oocyte enclosed by a single layer of flat pregranulosa (PG) cells, while an aggregation of two or more oocytes were defined as cysts ^[2]^. Oocytes were counted every 5 slices in successive section of each ovary as an independent biological replication.

**3. Immunohistochemistry (IHC)**

Serial sections of 21 dpp ovaries were obtained following the same procedure described above. After antigen retrieval, sections were treated as follows: incubation in 3% H_2_O_2_ for 10 minutes, blocking with 3% BSA and 10% normal goat serum in TBS for 1 hour, incubation with primary antibodies (Table S7) overnight at 4 ℃, washing with TBST, incubation with HRP-labeled secondary antibodies (Table S7) for 1 hour at 25 °C, treatment with DAB (ZSGB-BIO, ZLI-9017, Beijing, China) peroxidase substrate and counterstaining with hematoxylin.

For the estimation of different classes of follicles in 21 dpp ovaries, we classified primordial and primary follicles as those containing an intact MVH-positive oocyte surrounded by a single layer of flat or cuboidal granulosa cells. Secondary follicles were characterized by having two or multiple layers of granulosa cells, while antral follicles were identified by the presence of a fluid-filled cavity adjacent to the oocyte. The total number of each follicle class were counted every 5 slices in successive section of each ovary as an independent biological replication.

**4. ROS measurement**

ROS amount was measured in ovaries fresh collected from WT and *Cacna2d3* KO mice using ROS Assay Kit (Beyotime, S0033, Shanghai, China). Briefly, ovaries were incubated with 10 μM DCFH-DA for 15 minutes at 37 °C in the dark, thoroughly washed and photographed at the same exposure time under a fluorescence microscope using ImageJ software (NIH, Bethesda, MD, USA).

**5. Glutathione (GSH) and oxidized GSH (GSSG) ratio measurement**

Total GSH and GSSG levels were determined using GSH and GSSG assay kits (Beyotime, S0053). Briefly, ovarian lysates from WT and *Cacna2d3* KO were obtained following the manufacturer's guidelines, and divided into two parts for total GSH and GSSG measurements; GSH/GSSG was defined as (total GSH - GSSG) / GSSG.

**6. Western blot analysis**

Ovarian protein samples were obtained using RIPA lysates (Beyotime, P00113B). Western blot (WB) was performed using standard procedures: Briefly, after separation by sodium dodecyl sulfate-PAGE, the gel was transferred to the polyvinylidene fluoride membrane (Millipore, ISEQ00010, USA) and blocked at 25 °C for 2 hours. The membrane was then incubated with primary antibodies (Table S7) for half a day and after three washes with HRP-conjugated secondary antibodies (Table S7) for 1 hour. Chemiluminescence detection was performed using a BeyoECL plus Kit (Beyotime, P0018), and the AlphaView SA software (ProteinSimple, San Jose, CA, USA) was used to calculate the relative intensities of protein bands.

**7. RNA extraction and real time quantitative PCR (RT-qPCR)**

Total ovarian RNA was extract with the prep pure Micro Kit (Aidlab, RN07, Beijing, China). cDNA synthesis kit (TransGen, AT311-03, Beijing, China) was used for reverse transcription according to the manufacturer’s instructions. RT-qPCR procedures were performed using the method previously reported ^[3]^. The primers are listed in Table S8, and the 2^−△△Ct^ method was used to determine mRNA expression levels.

**8. scRNA-seq sample preparation**

At least four *Cacna2d3* KO and WT female 3 dpp pups were used for scRNA-seq sample preparation. Whole ovaries were cut into small pieces in digestive solution containing 500 μl 2 mg/ml collagenase (Sigma-Aldrich, C5138, Shanghai, China) and digested at 37 °C for 5-10 min to obtain single cell suspension. Digestion was then terminated with a medium containing 10% serum and cell suspension was filtered with 40 μm filter to obtain single cells and washed with PBS for 3 times. Finally, cell viability and cell number were counted using a hemocytometer with trypan blue to meet the requirements of sequencing.

**9. Libraries preparation and scRNA-seq**

Single cell sample library preparation and sequencing were performed following the methods reported by Wang et al ^[4]^. Briefly, using the Single Cell 3’ Library and Gel Bead Kit V2 (10 × Genomics Inc., 120237, Pleasanton, CA, USA), samples were fed onto a 10× Chromium chip to generate single cell gel beads in emulsion (GEMs). According to the manufacturer’s protocol, scRNA-seq libraries were constructed with v3 chemistry (10× Genomics), and sequencing was carried out on a NovaSeq 6000 (Illumina). Cell Ranger v3.1.5 was used to generate output readings (150 bp pair-ended) using default parameters. Figure S1A shows the detailed information of collected cells for WT and *Cacna2d3* KO samples following the "count" flow.

**10. Clustering analysis using the Seurat package**

Raw data were filtered using R package Seurat V3.1.2 to remove low quality cells by analyzing mitochondrial percentages and genes in the sample. The R package DoubletFinder v2.0.3 was utilized to eliminate the influence of double cells ^[5]^. Meanwhile, cell clusters expressing two specific different cell markers were also examined and deleted in this experiment. Two objects were integrated through "FindIntegrationAnchors" and "IntegrateData" functions. Finally, visualization of cell clusters and discovery of specific marker genes were performed by “RunUMAP” function and “FindAllMarkers” function respectively.

**11. Pseudotime trajectory analysis using Monocle package**

The oocytes pseudotime trajectory analysis followed the methods reported by Tian et al ^[6]^. Briefly, the oocytes cluster and its highly variable genes identified by Seurat analysis were introduced into Monocle (v2.10) ^[7]^ for pseudotime trajectory analysis of oocytes by “reduceDimension” and “orderCells”. Finally, the BEAM function was utilized to determine the variation of branch specific gene expression around nodes in the pseudotime analysis results.

**12. Identification of DEGs**

In this experiment, DEGs for oocytes and PG cell of WT and *Cacna2d3* KO group were identified by the R package DEsingle analyses ^[8]^. DEsingle package can more properly address more zero values in scRNA-seq more reasonably. The screening threshold for identifying DEGs was *P*-value < 0.05.

**13. Functional enrichment and mapping**

Genetic Ontology (GO) analysis and the Kyoto Encyclopedia of Genes and Genomes (KEGG) were performed on DEGs sets using R packages “clusterProfiler” V3.10.1 ^[9]^. *P*-values less than 0.05 were deemed significant for GO keywords and signaling pathways. The online tools Metascape ^[10]^ were used to uncover the function GO terms of PG cell gene sets. In addition, gene set enrichment analysis (GSEA) ^[11]^ was used to reveal OXPHOS signaling pathways.

**14. Ligand-receptor pairs analysis between ovarian cells**

R packages CellChat ^[12]^ were used for ligand-receptor pair analysis between ovarian cells. Briefly, the number of inferred interactions and interaction strength of ovaries cell were compared between two group by configuring the CellChatDB database. “subsetCommunication” function was used to extract the ligand-receptor pairs between germ cells and PG cells, and “netVisual_chord_cell” and “netVisual_bubble” function was used for visual analysis.

**15. Ovary culture *in vitro* and siRNA interference**

Ovaries from 3 dpp pups were isolated and cultured as previously described ^[13, 14]^. Before transfection, 1.5 μL Transfection Reagent (TransGen Biotech, FT301, A) and 2 μL 20 μmol/L negative control (NC) siRNA (Sense: 5’-UUCUCCGAACGUGUCACGUTT-3’, Antisense: 5’-ACGUGACACGUUCGGAGAATT-3’) or *Kit* siRNA (Sense: 5’-GCUAACAAAGGGAAGGAUUTT-3’, Antisense: 5’-AAUCCUUCCCUUUGUUAGCTT-3’) (B) were added to 50 μL basal medium at room temperature for 5 min, respectively. Next, solution A was added to solution B for 15min, then transferred to 400 μL ovarian medium for transfection and replaced with fresh medium 8h later. Finally, the ovaries were collected for oocyte in cyst or follicle count after 3 days of culture.

**16. Statistical analysis**

All data are shown as the mean ± SD and GraphPad Prism 8 analysis software was used for data analysis. Unpaired two-tailed Student's t test was used to calculate the *P* value between the two groups of data.

**References**

[1]. Liu WX, Zhang YJ, Wang YF, et al. Protective Mechanism of Luteinizing Hormone and Follicle-Stimulating Hormone Against Nicotine-Induced Damage of Mouse Early Folliculogenesis[J]. Frontiers in cell and developmental biology, 2021, 9: 723388.

[2]. Pepling ME. From primordial germ cell to primordial follicle: mammalian female germ cell development[J]. Genesis, 2006, 44: 622-632.

[3]. Wang YF, Sun XF, Han ZL, et al. Protective effects of melatonin against nicotine-induced disorder of mouse early folliculogenesis[J]. Aging (Albany NY), 2018, 10: 463-480.

[4]. Wang JJ, Ge W, Zhai QY, et al. Single-cell transcriptome landscape of ovarian cells during primordial follicle assembly in mice[J]. PLoS Biol, 2020, 18: e3001025.

[5]. McGinnis CS, Murrow LM, Gartner ZJ. DoubletFinder: Doublet Detection in Single-Cell RNA Sequencing Data Using Artificial Nearest Neighbors[J]. Cell systems, 2019, 8: 329-337.e324.

[6]. Tian Y, Zhang MY, Zhao AH, et al. Single-cell transcriptomic profiling provides insights into the toxic effects of Zearalenone exposure on primordial follicle assembly[J]. Theranostics, 2021, 11: 5197-5213.

[7]. Trapnell C, Cacchiarelli D, Grimsby J, et al. The dynamics and regulators of cell fate decisions are revealed by pseudotemporal ordering of single cells[J]. Nature biotechnology, 2014, 32: 381.

[8]. Miao Z, Deng K, Wang X, et al. DEsingle for detecting three types of differential expression in single-cell RNA-seq data[J]. Bioinformatics, 2018, 34: 3223-3224.

[9]. Yu G, Wang L-G, Han Y, et al. clusterProfiler: an R package for comparing biological themes among gene clusters[J]. Omics: a journal of integrative biology, 2012, 16: 284-287.

[10]. Zhou Y, Zhou B, Pache L, et al. Metascape provides a biologist-oriented resource for the analysis of systems-level datasets[J]. Nature communications, 2019, 10: 1-10.

[11]. Yu G, Wang LG, Han Y, et al. clusterProfiler: an R package for comparing biological themes among gene clusters[J]. OMICS, 2012, 16: 284-287.

[12]. Jin S, Guerrero-Juarez CF, Zhang L, et al. Inference and analysis of cell-cell communication using CellChat[J]. Nat Commun, 2021, 12: 1088.

[13]. Liu WX, Donatella F, Tan SJ, et al. Detrimental effect of Bisphenol S in mouse germ cell cyst breakdown and primordial follicle assembly[J]. Chemosphere, 2021, 264: 128445.

[14]. Feng YQ, Wang JJ, Li MH, et al. Impaired primordial follicle assembly in offspring ovaries from zearalenone-exposed mothers involves reduced mitochondrial activity and altered epigenetics in oocytes[J]. Cell Mol Life Sci, 2022, 79: 258.

**Supplementary figures**


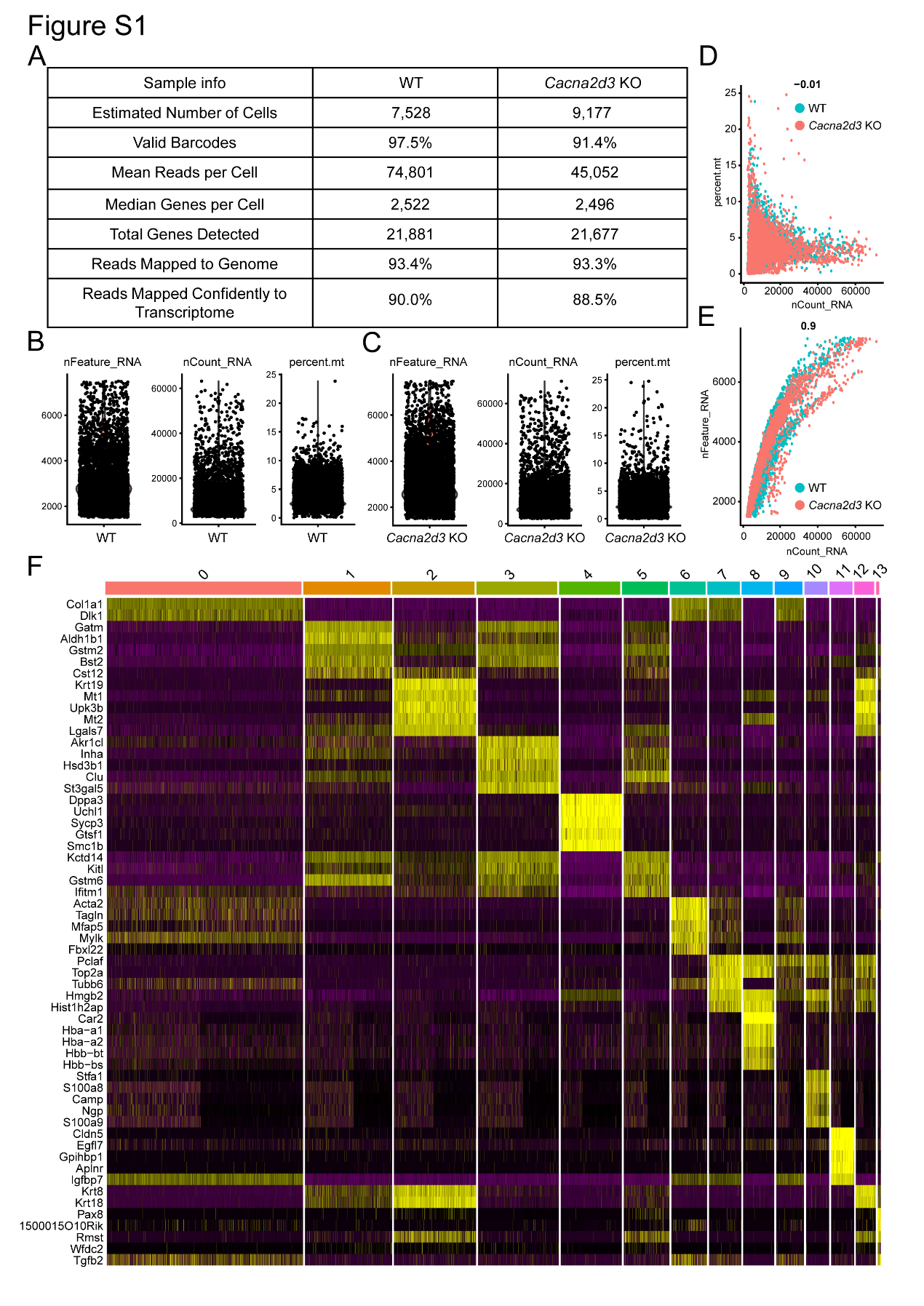


**Figure S1.** **scRNA-seq information and results quality control.**

(A) Statistics of sequencing data. (B and C) Violin plots showing the number of genes (nFeature_RNA), unique molecular identifier (nCount_RNA) and percentage of mitochondria genes (percent.mt) in the WT sets and *Cacna2d3* KO sets. (D) Scatter plot showing the correlation between nCount_RNA and percent.mt in the two ovary groups. (E) Scatter plot showing the correlation between nCount_RNA and nFeature_RNA in the two ovary groups. (F) Heatmap of the top 5 DEGs of each cell cluster.


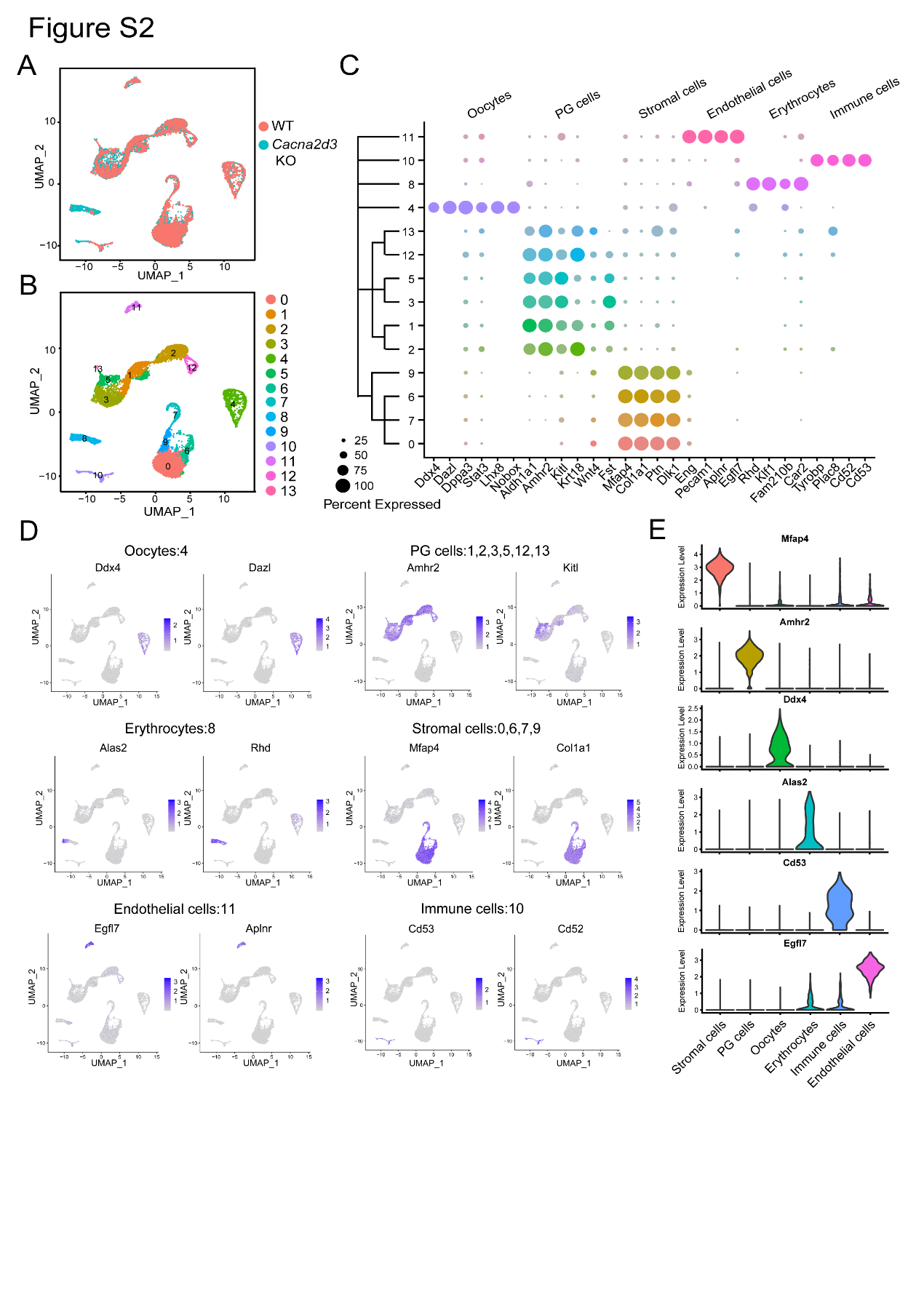


**Figure S2. Comprehensive characterization of ovarian cell types based on specific marker genes.**

(A) UMAP plot of ovarian cells based on sample grouped. (B) UMAP plot of ovarian cells based on Seurat cluster analysis. (C) Dot plot of the marker genes in the six main ovarian cell types. (D) Feature plots of specific marker genes from the six main ovarian cell types: Oocytes: *Ddx4*, *Dazl*; PG cells: *Amhr2*, *Kitl*; Erythrocytes: *Alas2*, *Rhd*; Stromal cells: *Mfap4*, *Col1a1*; Endothelial cells: *Egfl7*, *Aplnr*; Immune cells: *Cd53*, *Cd52*. (E) Violin plots of representative genes for oocytes, PG cells, stromal cells, erythrocytes, immune cells, and endothelial cells.


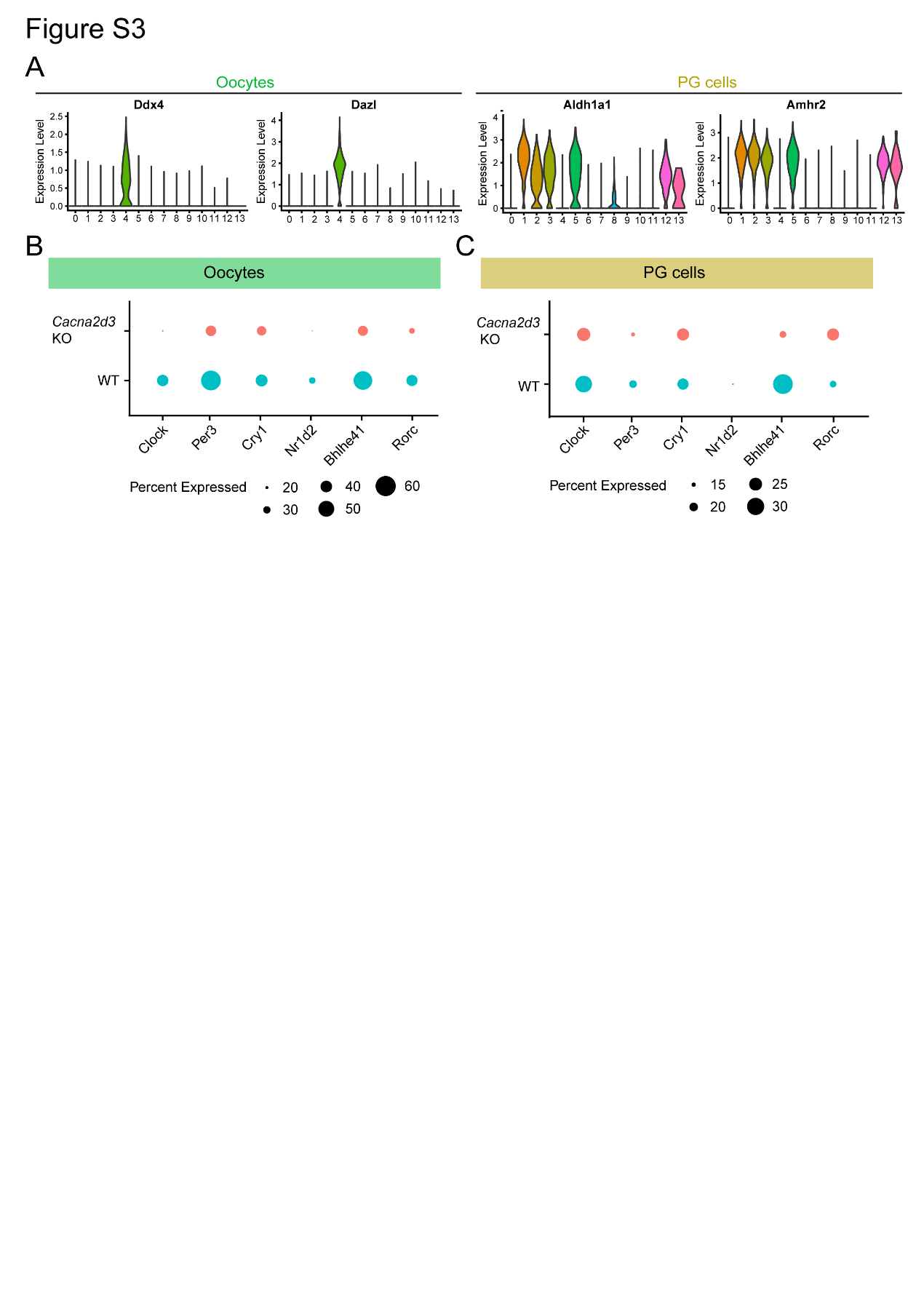


**Figure S3. Expression of cell** **specific marker and circadian clock genes in Oocytes and PG cells.**

(A) Violin plots of specific marker gene in oocytes (*Ddx4*, *Dazl*) and PG cells (*Aldh1a1*, *Amhr2*). (B) Bubble plots showing the expression levels of circadian clock genes in oocytes from WT and *Cacna2d3* KO 3dpp ovaries. (C) Bubble plots showing the expression levels of circadian clock genes in PG cells from WT and *Cacna2d3* KO 3dpp ovaries.


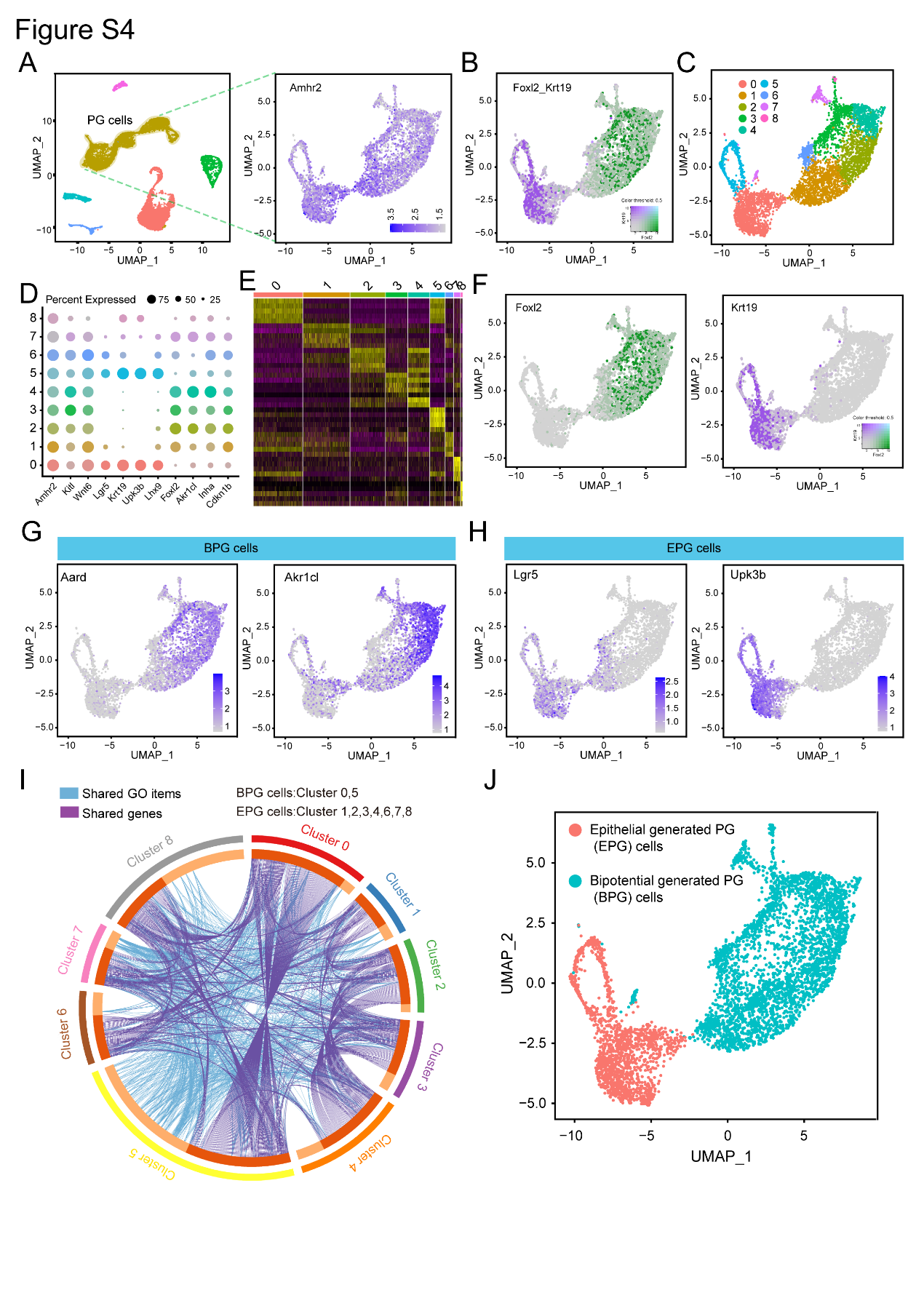


**Figure S4**. **Characterization and subdivision of PG cell clusters in ovarian.**

(A) Extraction of *Amhr2*-positive PG cell cluster in the UMAP map. (B) PG clusters were separated on the base of expression of *Foxl2* and *Krt19*. (C) UMAP of the PG cell cluster subdivided in 9 subclusters. (D) Dot plot showing the percent expression of the marker genes in the 9 subclusters. (E) Heatmap of the top 5 DEGs of each 9 PG cell subclusters. (F) Expression of *Foxl2* and *Krt19 in the* PG cell clusters. (G) Expression of *Aard* and *Akr1cl* identify BPG cells; color intensity indicates the expression level. (H) Expression of *Lgr5* and *Upk3b* identify EPG cells; color intensity indicates the expression level. (I) Circos diagram showing DEGs and GO terms shared among the 9 PG cell clusters; purple line represents shared DEGs, blue line represents shared GO terms. (J) UMAP of the PG cell cluster subdivided in epithelial generated PG (EPG) and bipotential generated PG (BPG) clusters.


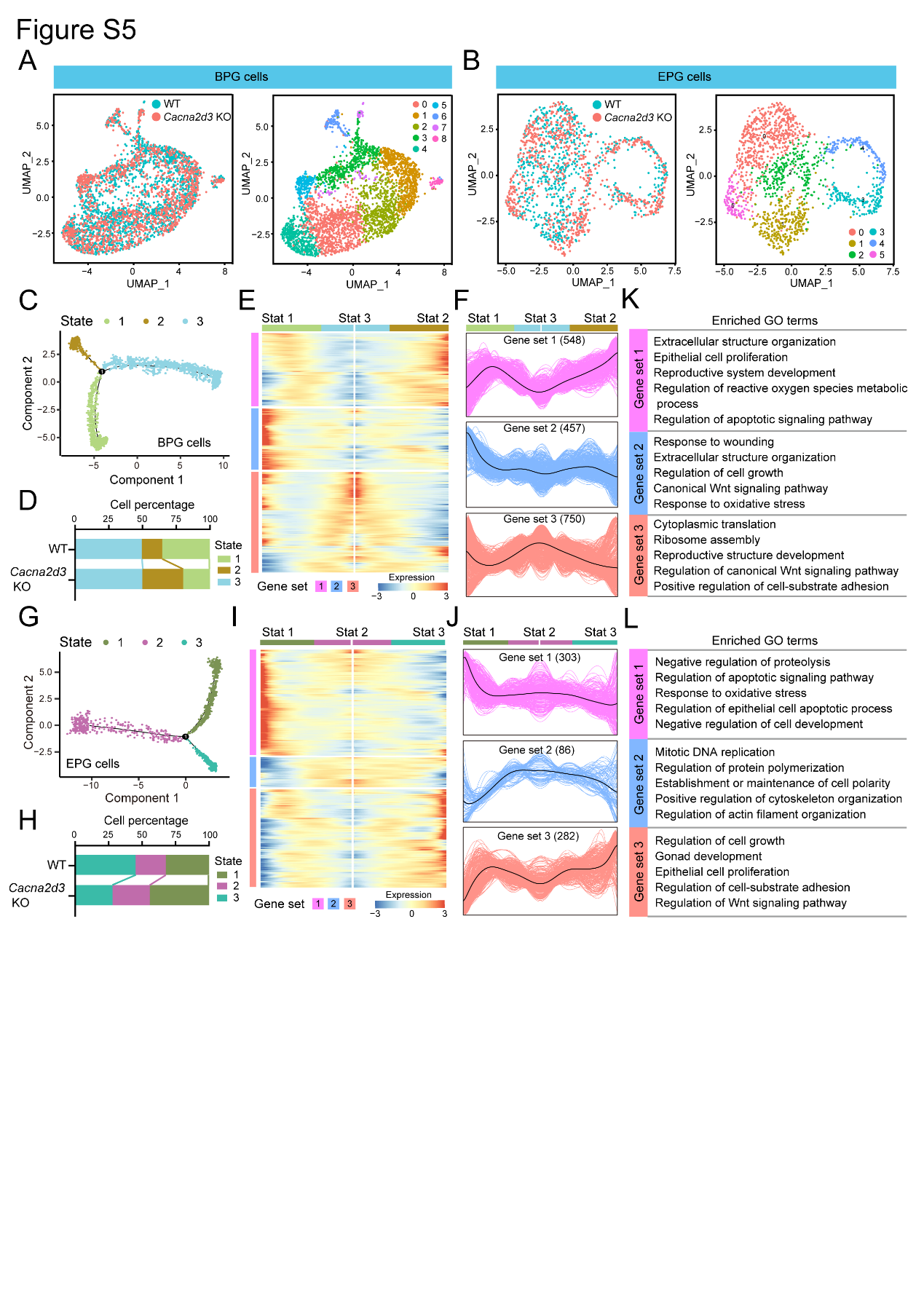


**Figure S5**. **Transcriptome differences between WT and *Cacna2d3* KO PG cells from 3dpp ovaries.**

(A) UMAP plot of WT and KO BPG cells (left) and their clusters (right). (B) UMAP plot of WT and KO EPG cells (left) and their clusters (right). (C) Single-cell pseudotime developmental trajectory of the BPG cells populations showing 3 states and one branching point. (D) Percent distribution of the BPG cells into the 3 states. (E) Pseudotime ordered heatmap of three DEGs sets between two obvious fates from State 3 to State 1 or State 2. (F) Graph showing the expression of the three DEGs sets in the pseudotime trajectory. (G) The enrichment of GO terms in gene set 1, 2, 3. (H) Single-cell pseudotime developmental trajectory of the EPG cells populations showing 3 states and one branching point. (I) Percent distribution of the EPG cells into the 3 states. (J) Pseudotime ordered heatmap of three DEGs sets between two obvious fates from State 2 to State 1 or State 3. (K) Graph showing the expression of the three DEGs sets in the pseudotime trajectory. (L) The enrichment of GO terms in gene set 1, 2, 3.


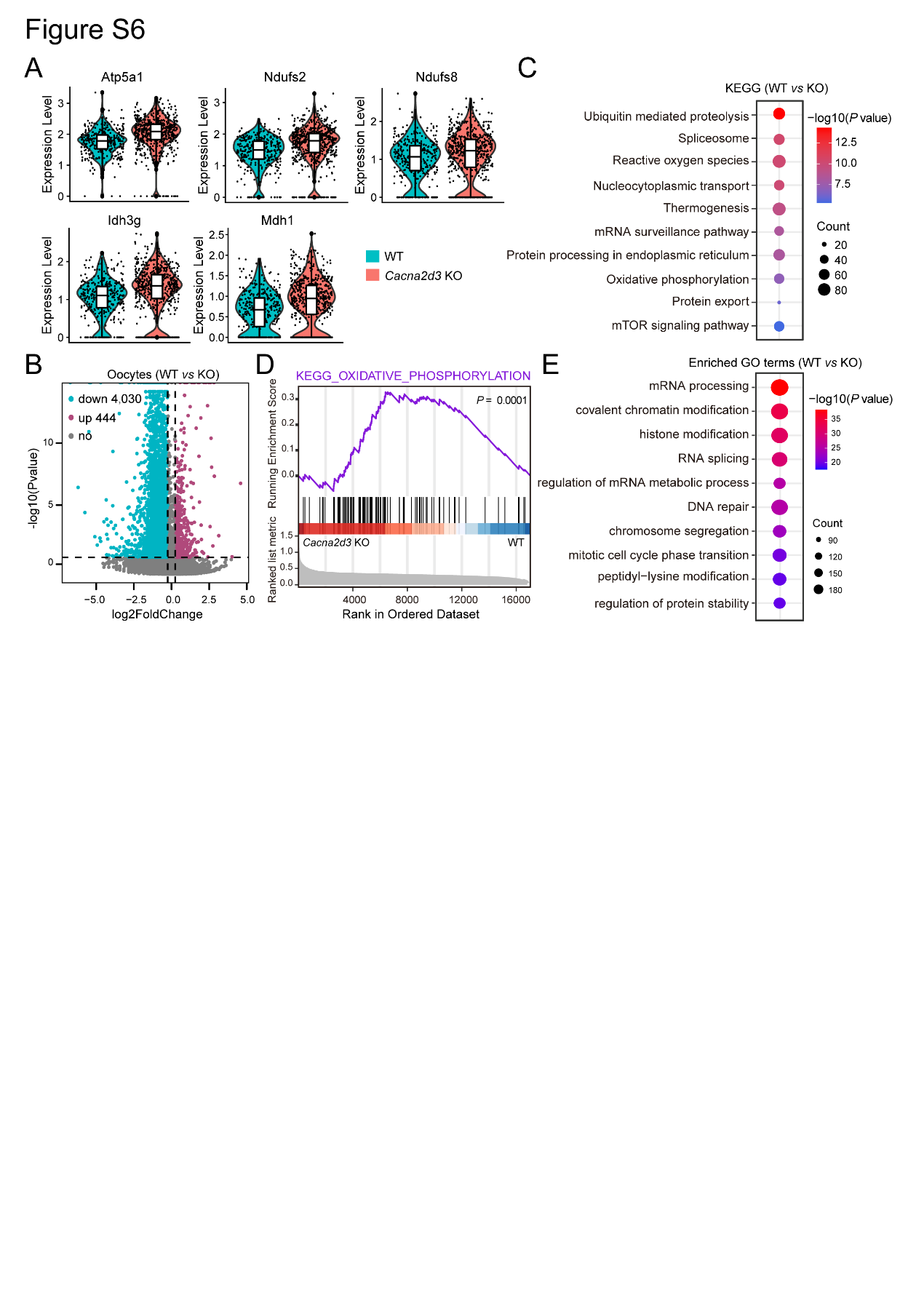


**Figure S6**. **Activation of OXPHOS signaling pathway in *Cacna2d3* KO oocytes.**

(A) Violin plots of the expression level of energy metabolism-related genes in oocytes of WT and *Cacna2d3* KO mice. (B) The volcano plot showing the DEGs of oocytes between WT and *Cacna2d3* KO mice. (C) Bubble chart shows the top 10 KEGG enrichment results of DEGs in oocytes of WT and *Cacna2d3* KO mice. (D) GSEA of the OXPHOS signaling pathway of oocytes. (E) Bubble chart shows the top 10 GO enrichment results of DEGs in oocytes of WT and *Cacna2d3* KO mice.


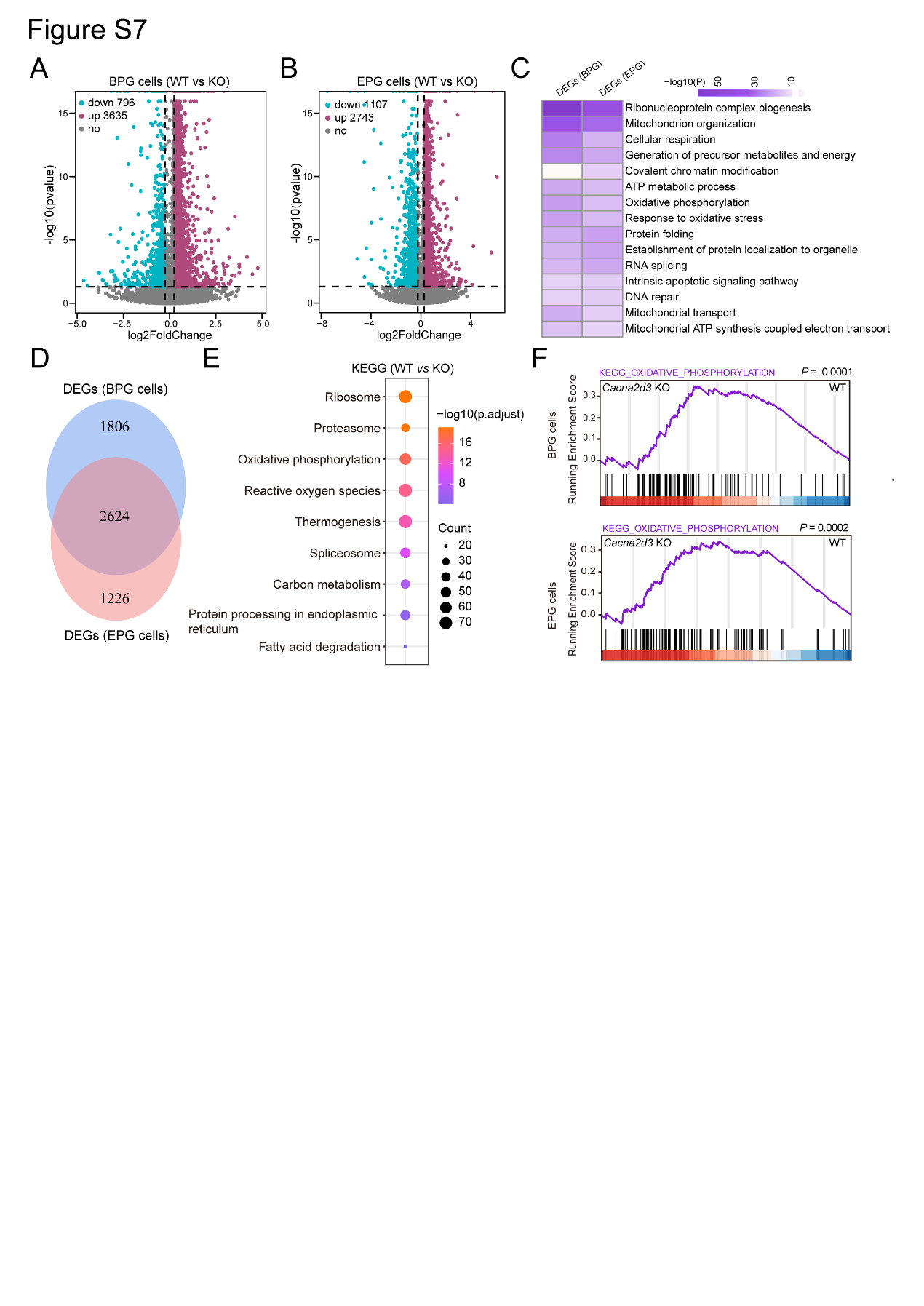


**Figure S7**. **Activation of OXPHOS signaling pathway in *Cacna2d3* KO PG cells.**

(A) Volcano plot showing DEGs between WT and KO BPG cells. (B) Volcano plot showing between WT and KO EPG cells. (C) Heatmap of GO enrichment terms for DEGs in BPG and EPG cells. (D) Venn diagram of DEGs between BPG and EPG cells. (E) Bubble chart showing KEGG enrichment terms of DEGs shared between BPG and EPG cells. (F) GSEA of OXPHOS signaling pathway in BPG and EPG cells.


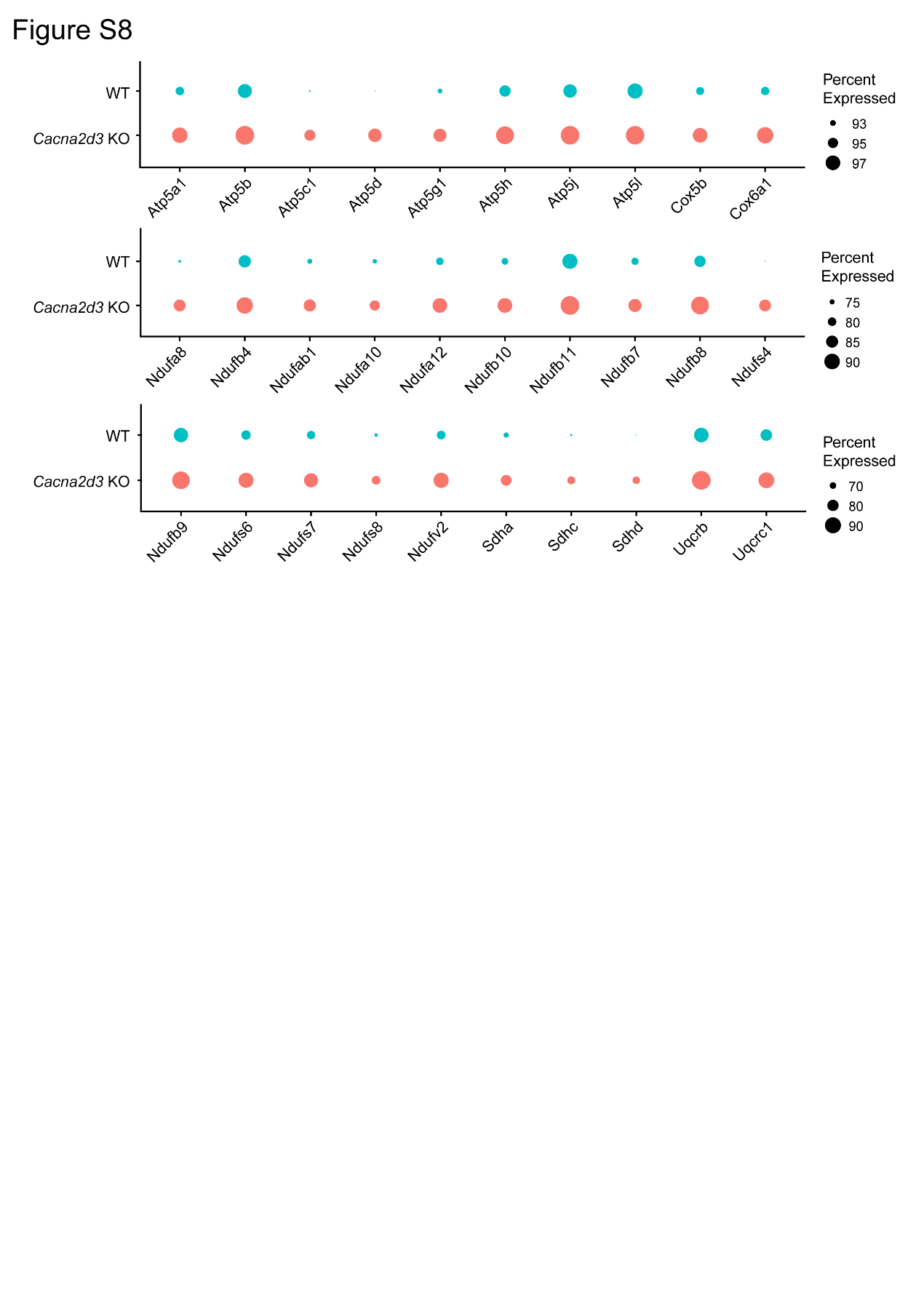


**Figure S8. Bubble plots showing the expression level of DEGs for OXPHOS signaling pathway in WT and Cacna2d3 KO 3dpp ovaries.**


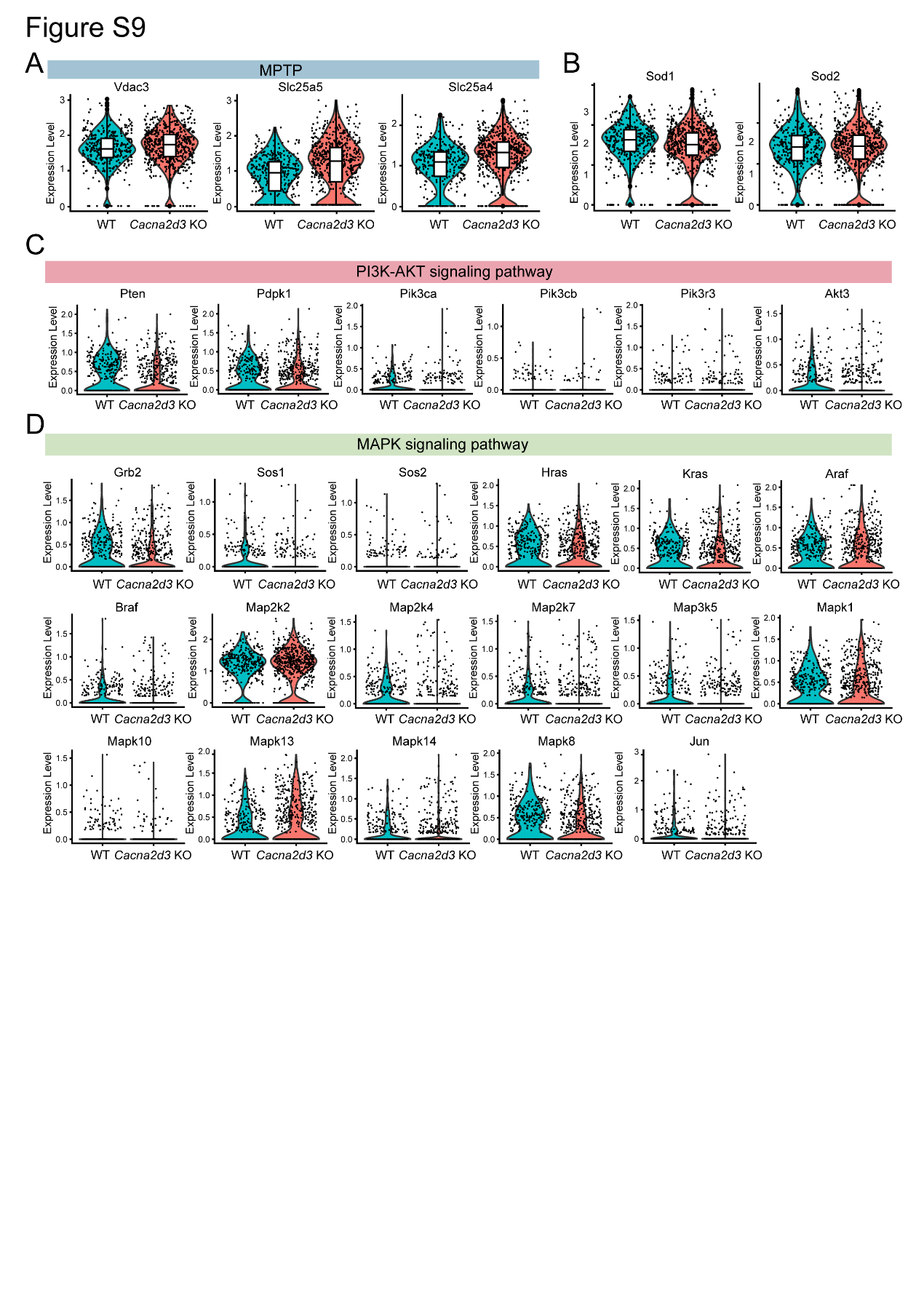


**Figure S9. Violin plots showing the expression level of (A) Mitochondrial permeability transition pore (MPTP)-related genes, (B) SOD-related gens, (C) PI3K-AKT signaling pathway genes, (D) MAPK signaling pathway genes, in WT and Cacna2d3 KO 3dpp ovaries.**


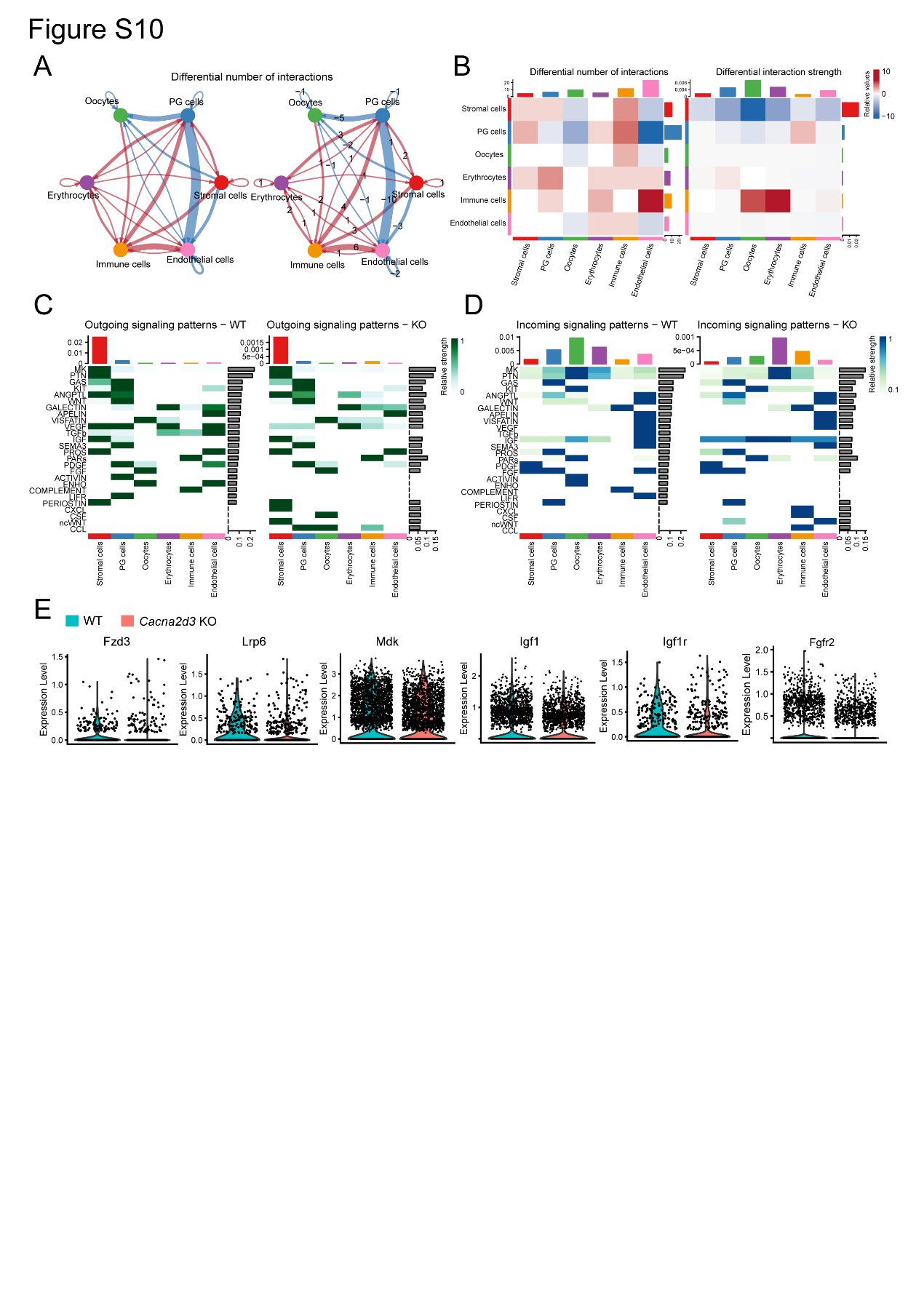


**Figure S10. Effect of *Cacna2d3* KO on communications among the main six cell populations of the 3dpp ovaries.**

(A) Differential number of interactions in ovaries of WT mice and *Cacna2d3* KO mice; Red and blue indicate increase or decrease, respectively, in KO compared with WT ovaries; thickness indicates the number of interactions. (B) Heatmap showing differential number of interactions (left) and differential interaction strength (right) in WT and *Cacna2d3* KO ovaries. (C and D) Heatmap showing outgoing and incoming signaling patterns in WT and *Cacna2d3* KO ovaries. (E) Violin plots representing the expression level of differential ligands and receptors in WT and *Cacna2d3* KO ovaries.


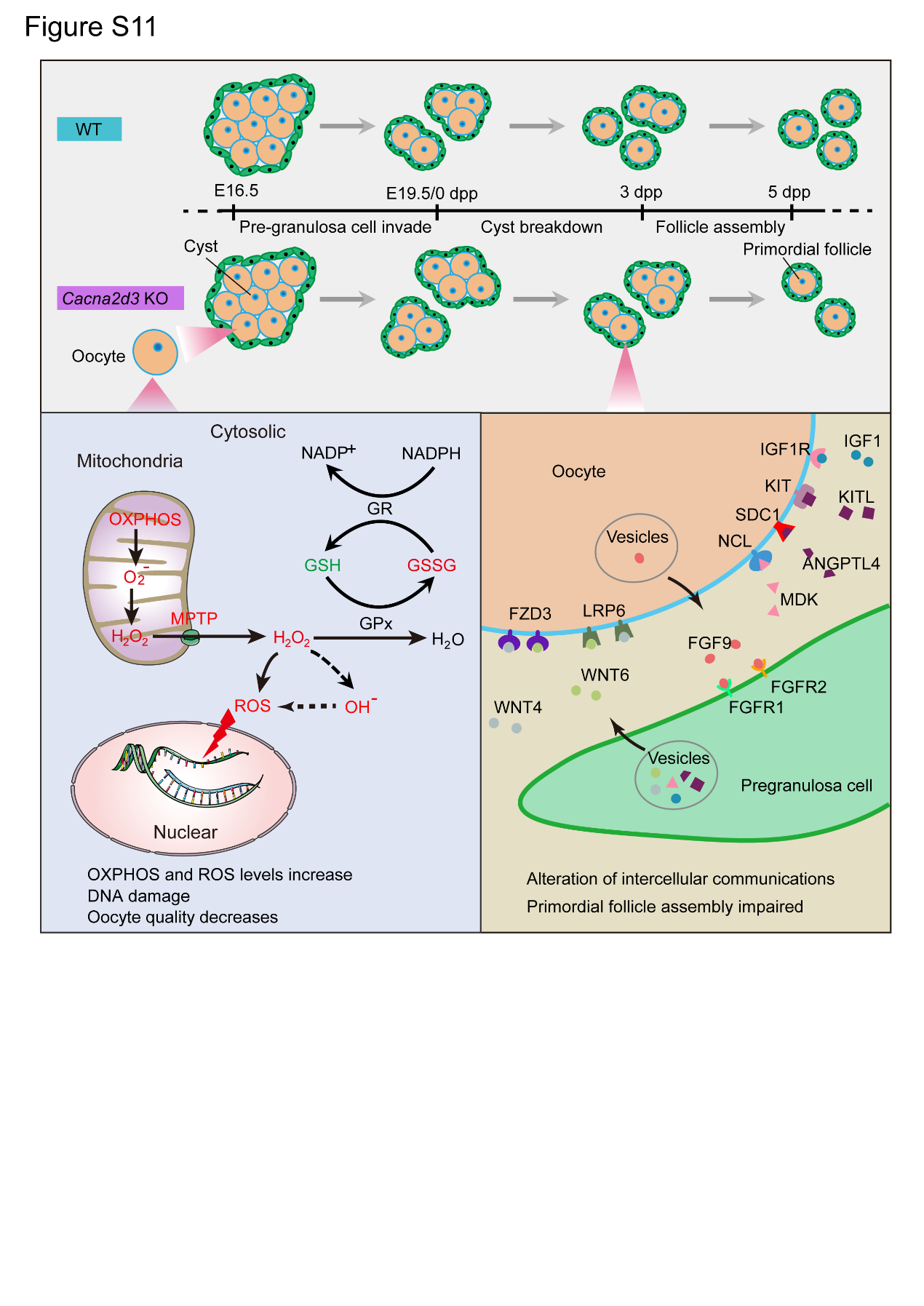


**Figure S11. Schematic drawing of the main changes occurring in the oocyte cysts as the result of *Cacna2d3* KO leading to reduced PF stockpile in the ovary.**

Upper panel indicates the time window of PF formation. Lower left panel indicates altered transcriptome of OXPHOS signaling pathway leading to increased ROS generation in mitochondria and consequent DNA damage and reduced oocyte quality. Lower right panel shows impairment of ligand-receptor pairs between oocyte and PG cells crucial for PF assembly.
